# ERManI Is a Target of miR-125b and Promotes Transformation Phenotypes in Hepatocellular Carcinoma (HCC)

**DOI:** 10.1371/journal.pone.0072829

**Published:** 2013-08-05

**Authors:** Shujuan Pan, Xiaoyun Cheng, Hongan Chen, Patricia D. Castro, Michael M. Ittmann, Anne W. Hutson, Susan K. Zapata, Richard N. Sifers

**Affiliations:** 1 Department of Pathology & Immunology, Baylor College of Medicine, Houston, Texas, United States of America; 2 Department of Molecular and Cellular Biology, Baylor College of Medicine, Houston, Texas, United States of America; 3 Department of Molecular Physiology and Biophysics, Baylor College of Medicine, Houston, Texas, United States of America; 4 Department of Pediatrics-Gastroenterology, Hepatology & Nutrition, Baylor College of Medicine, Houston, Texas, United States of America; 5 Department of Lymphoma & Myeloma, University of Texas-M D Anderson Cancer Center, Houston, Texas, United States of America; 6 Participant in the Baylor College of Medicine Summer Medical and Research Training Program, Baylor College of Medicine, Houston, Texas, United States of America; The University of Hong Kong, China

## Abstract

The MAN1B1 gene product, designated ER alpha-1, 2-mannosidase (ERManI), is an enzyme localized in the Golgi complex of mammalian cells. By functioning as a “gate keeper” to prevent the inappropriate secretion of misfolded glycoproteins, it plays a critical role in maintaining protein homeostasis in the mammalian secretory pathway. In the present study, we identified that a conserved motif within the 3’UTR of ERManI is a target of miR-125b, a microRNA frequently down-regulated in numerous types of cancers, including hepatocellular carcinoma (HCC). As predicted, the expression of ERManI is significantly elevated in HCC, as measured by immunohistochemistry in a liver spectrum tissue microarray. Additional analyses using several hepatoma cell lines demonstrated that the elevated ERManI inversely correlates with a diminished intracellular concentration of miR-125b. Moreover, functional studies indicated that RNAi-mediated knock-down of endogenous ERManI was sufficient to inhibit proliferation, migration, and invasion of hepatoma cells. These phenotypical changes occurred in the absence of alterations in global glycoprotein secretion or ER-stress status. Together, these results revealed a novel post-transcriptional regulatory mechanism for ERManI and implied that this molecule contributes to the regulation of carcinogenesis in HCC independent of its function in glycoprotein quality control.

## Introduction

Hepatocellular carcinoma (HCC) is the sixth most common cancer and the third largest cause of cancer-related death world-wide [1–3]. The rising incidence of HCC demands more efficient strategies for therapeutic interventions, which will be based on a thorough understanding of the etiology of the disease. However, despite the discovery of many molecular mechanisms that induce hepatocarcinogenesis, our understanding about the exact mechanisms that lead to uncontrolled cell proliferation and migration of hepatoma cells is still limited [4].

miRNAs are small endogenous single stranded, non-coding RNAs consisting of 20-22 nucleotides. They function through binding to specific sequences at the 3’UTR of target mRNAs, which lead to either translational repression or degradation of the target transcript [5]. Ample evidence now demonstrates that miRNAs are among the key regulatory molecules of nearly every cellular process, including cell proliferation, differentiation and programmed cell death [6–8]. Alterations in miRNA expression contribute to the pathogenesis of many types of diseases including cancer [9–13]. In HCC, the aberrant expression of many miRNAs has been reported in cancerous tissues [14–19]. In particular, downregulation of miR-125b has been discovered by several groups as a signature event for HCC [14,20], and this single miRNA can provide predictive significance for prognosis in HCC patients [15]. Importantly, ectopic expression of miR-125b inhibits the proliferation, invasion and tumorigenesis potential of liver cancer cells [21,22], suggesting its tumor suppressor role in liver cancer. Despite these findings, the exact roles for miR-125b downregulation in hepatocarcinogenesis remain largely unclear.

Human endoplasmic reticulum mannosidase I (ERManI) is a type II transmembrane protein predominantly localized to the Golgi apparatus [23]. This molecule is known as a protein quality control factor that helps distinguish misfolded N-linked glycoproteins for proteasome-mediated degradation [24–26]. By doing so, ERManI is predicted to alleviate endoplasmic reticulum stress (ER-stress) imposed by the accumulation of misfolded proteins in the secretory pathway, which contributes to the global cellular protein homeostasis [27]. In yeast, a null mutation in the ERManI ortholog, designated MNS1, inhibits the degradation of misfolded glycoproteins such as CPY* [28]. In mammalian cells, siRNA-mediated knockdown of ERManI in the human carcinoma cell line HeLa or embryonic kidney cell line 293 inhibits the degradation of several misfolded glycoproteins such as mutant HA and alpha-1 antitrypsin (A1AT) [23,29,30]. Recently, we observed that ERManI physically associates with mutant A1AT. Knockdown of endogenous ERManI in HeLa cells not only leads to intracellular accumulation of transfected recombinant mutant A1AT, but it also promotes secretion of the substrate, implying that ERManI is capable of contributing to a complex that captures newly synthesized misfolded proteins that escape to the Golgi complex [31].

The intracellular concentrations of most protein quality control components are transcriptionally regulated, the induction of which can promote the eventual resolution of ER stress [32,33]. In contrast, the concentration of the endogenous ERManI mRNA is not elevated even during acute ER-stress [33]. Several studies have shown that ERManI is mainly regulated by post-transcriptional mechanisms [34–36]. In particular, we have identified a single nucleotide polymorphism in the 3’-untranslated region (3’-UTR) of the corresponding gene (*MAN1B1*) that encodes human ERManI. Homozygosity for the resulting hypomorphic allele, which suppresses the translation of the encoded mannosidase, contributes to the early onset of end-stage liver disease possibly by impairing the intracellular disposal of alpha-1 antitrypsin [35]. This finding has prompted us to investigate the translational regulation of ERManI via the 3’UTR of its transcript. In the present study, we determined that the ERManI 3’-UTR is a target of miR-125b, which is known to be down-regulated in HCC [14,15,20]. The depletion of miR-125b in hepatocellular carcinomas (HCC), in combination with a concomitant elevation in endogenous ERManI expression, coincided with enhanced cell proliferation, migration, and invasion.

## Results

### ERManI is targeted by miR-125

**Figure 1:**
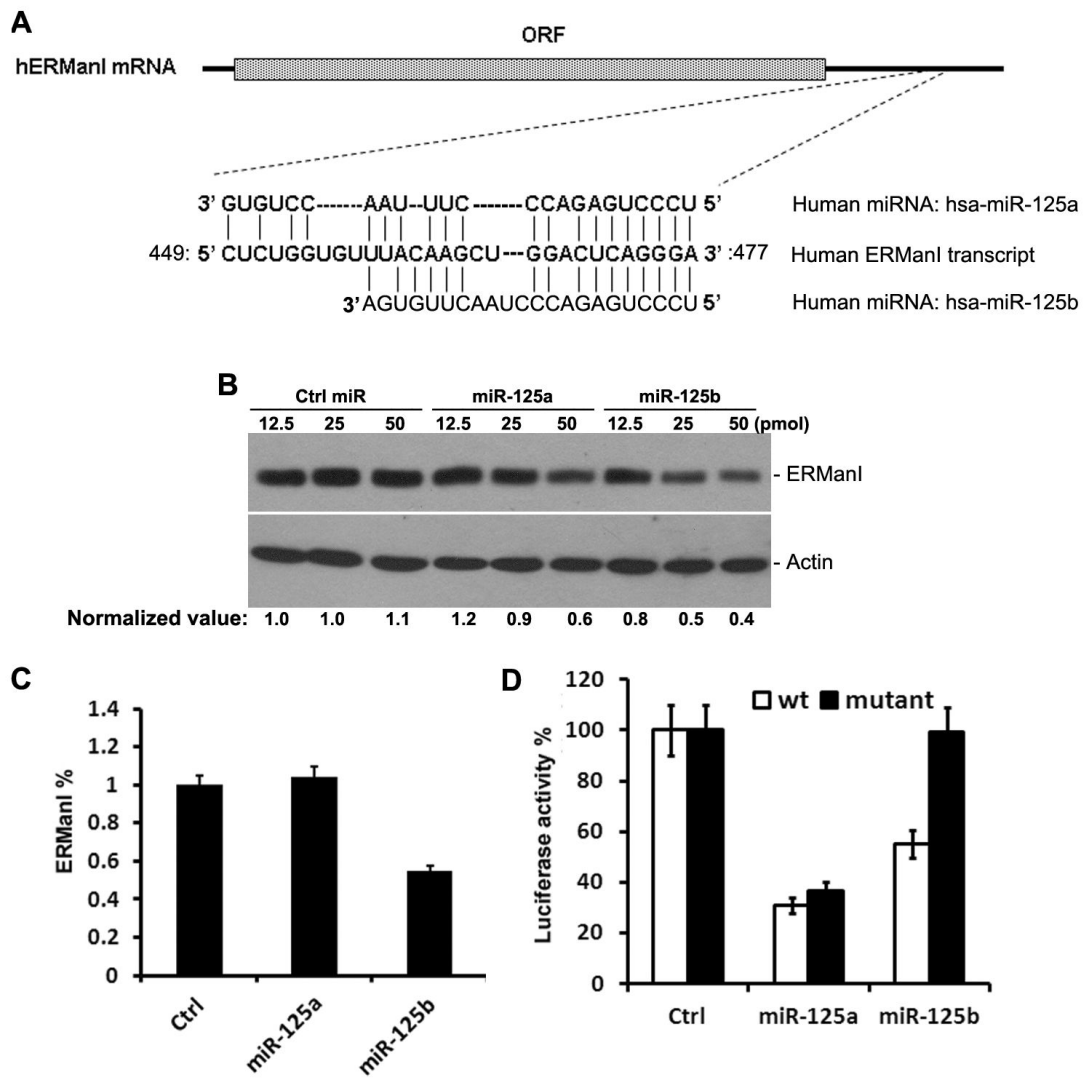
ERManI is a target of miR-125b. **A**. Illustration of the predicted target sequence of miR-125a and miR-125b located at the 3’-UTR of ERManI mRNA. “CUCAGGGA” on ERManI transcript represents the seed sequence. **B**. Western blotting of ERManI and actin (used as loading control) in PLC/PRF5 cells transfected with indicated amounts of control miRNA, miR-125a, or miR-125b. **C**. The percentage of ERManI expressed in MCF7 cells transfected with 12.5pmol miR-125a or miR-125b as compared to that transfected with equal amounts of control miRNA. The data were based on the densitometry measurement of the protein bands detected by western blotting. Error bars represent one standard deviation from three repeated samples. **D**. The luciferase constructs carrying the wild-type 3’UTR of ERManI or the ERManI 3’UTR with a mutated seed region were transfected into HeLa cells, and the luciferase activities were measured 24hr after transfection. The average activities from three repeated samples were used to calculate the percentage of inhibition. Error bars represent the standard error of the mean from three independent experiments.

The human ERManI transcript contains a ~600bp 3’-UTR. To explore the possible regulation of ERManI by miRNAs, we performed *in silico* analysis of miRNAs predicated to target the 3’UTR of its transcript. Several online softwares, including PicTar, TargetScan, and microrna, predicted that the sequence between nucleotides 449 and 477 is likely targeted by both miR-125a and miR-125b (Figure 1A). However, no validation for these predictions has been published.

To determine whether ERManI is a *bona fide* target of miR-125, we transfected control, miR-125a, or miR-125b mimics at different concentrations into PLC/PRF5 cells, a hepatoma cell line that expresses high levels of ERManI, and examined the changes in ERManI expression by western blotting. As compared to the effects of control siRNAs, both miR-125a and miR-125b inhibited the expression of ERManI (Figure 1B). However, while transfection with miR-125b downregulated ERManI expression to 80%, 50%, and 40% with 12.5pmol, 25pmol, and 50pmol miRNA mimics, respectively, miR-125a did not lead to a significant change in the ERManI level until the miRNA mimic reached 50pmol. These results indicated that ERManI can be regulated by miR-125 family members, but most potently by miR-125b.

We next tested whether the function of miR-125 in regulating ERManI is cell type-specific. For this, a breast cancer cell line MCF7 was used because of its high expression of protein. As shown in Figure 1C, at a concentration of 12.5pmol, miR-125b also showed an average of 50% suppression of ERManI, while miR-125a did not show significant effects (Figure 1C). ~60% inhibition of ERManI was reached by miR-125a, but required a concentration higher than 50pmol (Figure S1). This result further confirmed that miR-125b is more potent than miR-125a in suppressing ERManI expression, and this effect is cell type-independent.

To confirm whether miR-125a and miR-125b target the predicted sequence at the 3’UTR of ERManI, a luciferase reporter containing the wild-type 3’UTR of ERManI was generated. Using this construct as a backbone, the GG nucleotides (underlined sequence in Figure 1A) at the “seed” region (CUCAGGGA) of the predicted binding site was mutated to AA. The wild-type and mutant luciferase reporters were transfected separately into HeLa cells in combination with 50pmol of control miRNA, miR-125a, or miR-125b. 48hr post-transfection, the luciferase activities were measured. As shown in Figure 1D, both miR-125a and miR-125b showed significant inhibition of the luciferase activity when the wild-type 3’-UTR was present. However, only mutations at the seed region inhibited the function of miR-125b completely, but not that of miR-125a. This result indicated that miR-125b, but not miR-125a, targets the predicted binding sequence.

### Inverse expression of miR-125b and ERManI

**Figure 2:**
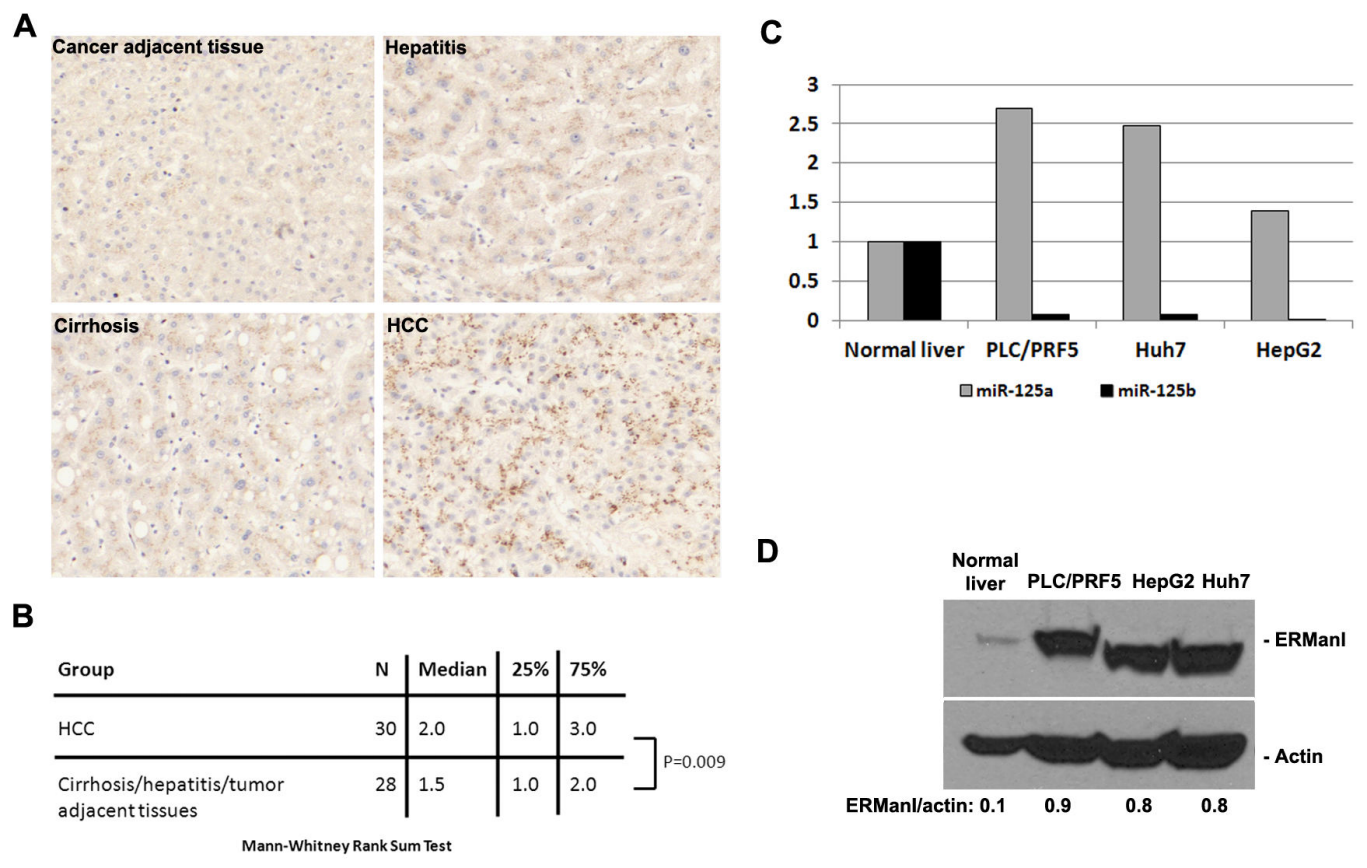
ERManI is upregulated in HCC. **A**. Representative images of different liver tissues stained with anti-ERManI antibodies. **B**. Statistical analyses of the HCC and cirrhosis/hepatitis/tumor adjacent tissues using Mann-Whitney Rank Sum Test. The analyses were based on the sample number (N), median, and intensity score at 25% and 75% percentile of each indicated tissue group. **C**. The percentage of miR-125a and miR-125b is expressed in PLC/PRF5, Huh7, and HepG2 cells lines as compared to that expressed in normal liver tissues. The data are based on the results obtained from real-time RT-PCR. **D**. Western blotting of ERManI and actin in normal liver and indicated cell lines. Numbers on the bottom represent the relative expression of ERManI normalized against actin.

miR-125b is significantly downregulated in HCC [14,15,20]. As a *bona fide* target of miR-125b, ERManI is likely to be upregulated. To test this possibility, we performed immunostaining of ERManI on a liver spectrum tissue microarray that contains 80 tissue samples ranging from normal hepatic tissue, cancer adjacent hepatic tissue, HCC, cholangiocellular carcinoma, cirrhosis, and hepatitis. A previously well-characterized anti-ERManI monoclonal antibody, 1D6, was used for staining. The results were analyzed by an experienced pathologist and the intensity score (IS) and percentage score (PS) given to each sample (Table S1) were used for statistical analysis. As compared to other groups of tissues, HCC showed a much higher intensity of ERManI staining (Figure 2A). By the Mann-Whitney rank sum test, the intensity of ERManI was significantly higher in HCC than in other groups (P=0.009) (Figure 2B), confirming that ERManI is indeed upregulated in HCC.

To obtain more evidence about the reverse correlation between miR-125b and ERManI in HCC, we compared the levels of these two molecules expressed in hepatoma cell lines to those expressed in normal liver. First, total RNAs were extracted from three hepatoma cell lines (PLC/PRF5, HepaG2, and Huh7), as well as normal human liver tissue, and the expression of miR-125b was determined by real time RT-PCR. In parallel, the expression of miR-125a was also examined. As shown in Figure 2C, miR-125b showed > 90% decrease and was barely detectable in three cell lines. In contrast, the miR-125a level in the three cell lines were 0.5~2.5-fold higher than that in the normal liver. This result not only confirmed the downregulation of miR-125b in hepatoma cell lines, it also indicated that miR-125b and miR-125a are reversely regulated in HCC.

To determine whether ERManI expression is inversely correlated with that of miR-125b, we extracted total cellular proteins from the three cell lines, as well as from normal liver tissues used above, and evaluated the ERManI level using western blotting. As shown in Figure 2D, all three cell lines showed an 8~9-fold increase of ERManI as compared to normal liver tissue, inversely mirroring the expression of miR-125b. Together, these results demonstrated that ERManI is a target of miR-125b in HCC.

### ERManI is required for maintaining transformation phenotypes in hepatoma cell lines

**Figure 3:**
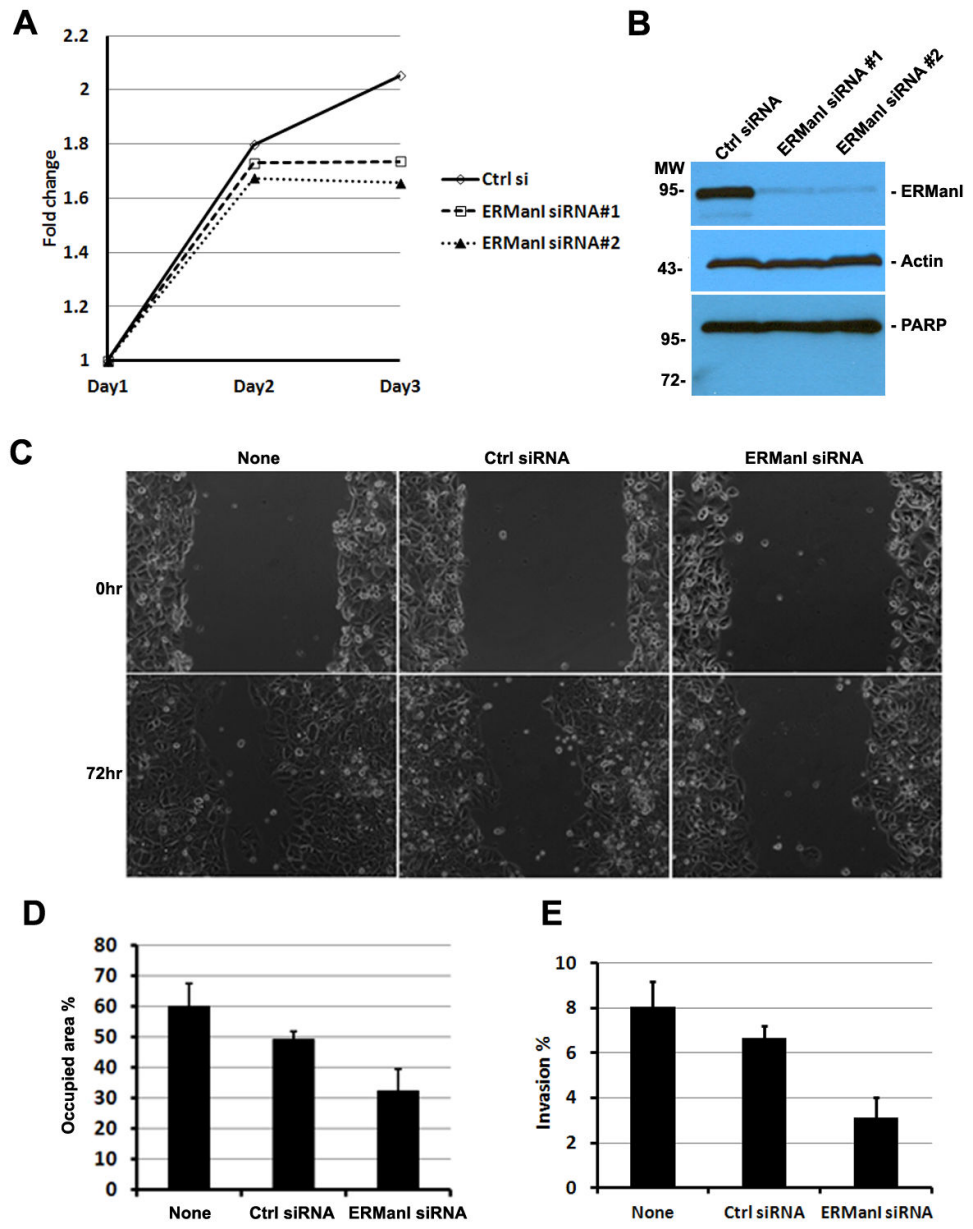
Downregulation of ERManI inhibits transformation phenotypes of PLC/PRF5 cells. **A**. Growth curve of PLC/PRF5 cells after transfection with control siRNA or two separate ERManI siRNAs #1 and #2 for 3days. **B**. Phase contrast images of non-transfected PLC/PRF5 cells or PLC/PRF5 cells transfected with control siRNA or ERManI siRNA at 0hr or 72hr after wound scratching. **C**. Average of the wounded areas occupied by non-transfected or siRNA-transfected PLC/PRF5 cells at 72hr post-scratching. Error bars represent standard deviations. **D**. Non-transfected PLC/PRF5 cells or PLC/PRF5 cells transfected with control siRNA or ERManI siRNA were subjected to the invasion assay, and the percentage of invaded cells were calculated against the total number of cells seeded. Error bards represent standard deviations from three repeated samples.

Cancer cells are characterized by high proliferation rates and enhanced migration/invasion [42]. To begin to understand the role of ERManI in HCC, we first evaluated the effects of ERManI downregulation on cell proliferation. For this, two siRNAs that specifically target ERManI were separately transfected into PCL/PRF5 cells and the growth rates were monitored for three days after transfection. Cells transfected with scrambled siRNAs were used as negative control. As shown in Figure 3A & B, a ~90% knockdown of ERManI by each of the two ERManI-specific siRNAs resulted in a decrease in cell numbers starting at day two post-transfection. By day three, a ~40% decrease was observed. The decrease in cell numbers upon ERManI knockdown was not due to an increase in apoptosis because an induction of PARP (Poly ADP-ribose polymerase) cleavage, a signature event during apoptosis [43–45], was not observed (Figure 3B). This result indicated that ERManI is required for efficient proliferation of hepatoma cells.

To determine whether ERManI plays any roles in regulating hepatoma cell migration/invasion, we transfected PLC/PRF5 cells with ERManI-specific siRNA and examined the cell behavior in wound healing and trans-well invasion assays. As shown in Figure 3C & D, the ability of cells transfected with ERManI-specific siRNAs to migrate toward the wound was significantly lower than that of non-transfected cells or cells transfected with control siRNAs. Consistently, when plated in the upper wells of the transwell plates, the number of cells that had migrated through the filter was significantly decreased after transfection with ERManI-specific siRNAs. Together, these results indicated that ERManI is required for migration/invasion of hepatoma cells.

### ERManI regulates transformation phenotypes independent of ER-stress

**Figure 4:**
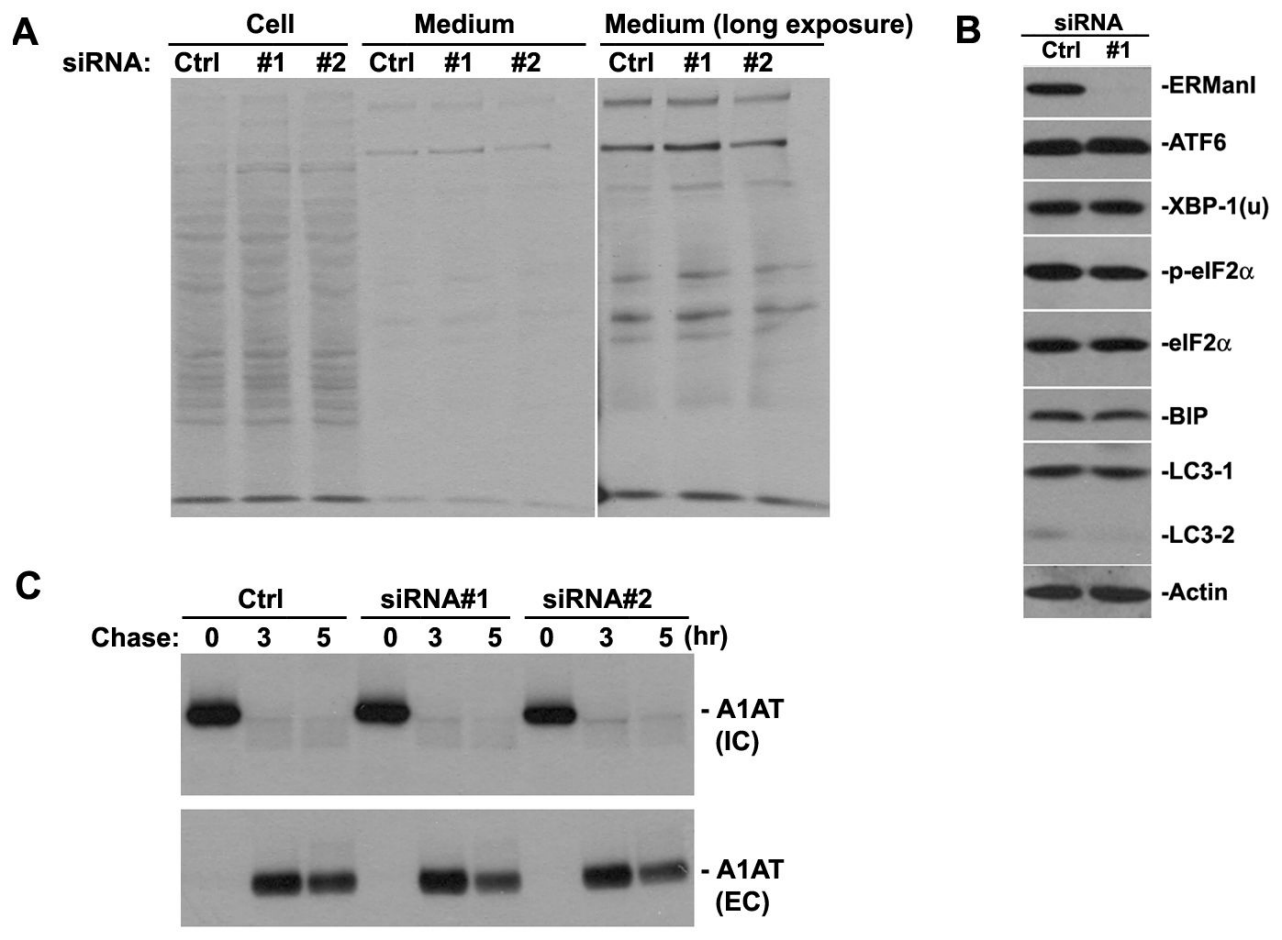
ERManI knockdown does not induce ER-stress. **A**. Autoradiography of total glycoproteins isolated from extracts and culture media of PLC/PRF5 cells after transfection with control siRNA or two separate ERManI siRNAs (#1 and #2). **B**. Western blotting of indicated the proteins in extracts of PLC/PRF5 cells following transfection with either control siRNA or ERManI-specific siRNA #1. **C**. PLC/PRF5 cells were transfected with either control siRNA (Ctrl) or two separate ERManI-specific siRNAs (#1 and #2). 48hr post-transfection, the cells were subjected to metabolic labeling with ^35^S-methionine and chased at 3hr and 5hr. The image shows the autoradiography of A1AT isolated from cell extracts (IC) or culture media (EC) of the PLC/PRF5 cells at indicated time points.

We have recently reported that ERManI functions as a “gate keeper” in the Golgi complex to facilitate the retention and recycling of misfolded glycoproteins escaped from the ER [31]. Lowering the endogenous level of ERManI allows misfolded glycoproteins to escape ERAD and be secreted [31]. It is possible that such effects will influence the overall intracellular level of glycoproteins, which subsequently changes the ER-stress status and indirectly affects cell proliferation and migration. As the first step to test this possibility, we used PLC/PRF5 cells to determine whether knockdown of endogenous ERManI affects overall glycoprotein secretion. For this, total glycoproteins were isolated from the ^35^S-labelled cells and media after transfection with a control siRNA or siRNAs targeting ERManI. The isolated proteins were analyzed using autoradiography. Unexpectedly, no significant changes in the overall amount of intracellular and extracellular glycoproteins were observed in the presence or absence of ERManI knockdown (Figure 4A), implying that the function of ERManI is selective, and does not affect the secretion of the majority of endogenous glycoproteins. Consistent with this notion, no significant differences in the level of major ER-stress markers, such as BiP, XBP-1, phosph-eIF2α, etc (Figure 4B) were detected, indicating that the overall ER-stress level remained the same upon ERManI knockdown. All together, these results support the notion that the function of ERManI in regulating cell transformation is independent of its function in regulating glycoprotein quality control.

### ERManI knockdown does not alter the modification and secretion of endogenous alpha-1 antitrypsin in PLC/PRF5 cells

The lack of effects resulting from ERManI knockdown on the global glycosylation pattern does not exclude the possibility that a small subset of glycoproteins might be affected. One of the best studied substrates of ERManI in protein quality control is alpha-1 antitrypsin (A1AT) [30]. In HCC, A1AT serves as one of the important diagnostic markers due to changes in the glycosylation status of the secreted protein [46–48]. We have previously reported that high levels of ERManI not only facilitate the degradation of misfolded AAT, but also influence the fate of wild type A1AT [30]. Therefore, it is possible that knockdown of ERManI in hepatoma cell lines could specifically affect the status of this protein. To explore this possibility, we knocked down ERManI in PLC/PRF5 cells and monitored the changes in the endogenous AAT using ^35^S-metabolic labeling experiments. As shown in Figure 4C, a ~60% knockdown of ERManI did not result in a dramatic change in either intracellular A1AT or extracellular A1AT at any time points examined. This observation negates the possibility that ERManI regulates transformation phenotypes of PLC/PRF5 cells via its influence on the biosynthetic maturation or secretion of A1AT.

## Discussion

ERManI is an important protein quality control component, but its role in cancer was never anticipated. In this study, we identified that, as a *bona fide* target of miR-125b, ERManI is highly expressed in HCC, and its knockdown inhibits proliferation and migration of hepatoma cells. These findings report, for the first time, the involvement of ERManI in cancer and reveal a novel connection between a protein quality control component and HCC.

This is the first study demonstrating the role of miRNA in regulating the ERManI concentration. However, although both miR-125a and miR-125b are predicted to target the transcript, only miR-125b targets the predicted sequence and can effectively suppresses ERManI expression. Because a much higher dose of miR-125a is required to suppress ERManI expression, it is likely that an indirect mechanism is actually involved. This notion is further supported by the fact that miR-125a does not target the predicted sequence, and the expression of miR-125a and ERManI are not inversely correlated. In contrast, miR-125b can potently inhibit the expression of proteins encoded by both ERManI cDNA and luciferase cDNA attached to the 3’-UTR of the ERManI transcript. In addition, mutation of the “seed region” of the predicted target sequence completely abolishes the inhibitory effect of miR-125b. Moreover, the expression of miR-125b and ERManI protein are inversely correlated in liver tissues and hepatoma cell lines. All these lines of evidence support the notion that ERManI is a *bona fide* target of miR-125b. miR-125b is known to regulate cell proliferation and stress responses, and its misregulation has been designated as a signature event in many types of cancers [13,49,50]. Therefore, it is predictable that, as a target of miR-125b, ERManI misregulation also occurs in cancers other than HCC. In fact, we have observed the reciprocal regulation of ERManI and miR-125b in several cancer cell lines (unpublished data), supporting this prediction. However, whether ERManI is targeted by miR-125b in non-cancerous cells remains to be determined.

The upregulation of ERManI in HCC was based on the immuno-staining of paraffin embedded liver tissue microarrays. This technique stains all the samples on a single slide, and therefore avoids technical variations which allows the entire cohort of samples to be stained uniformly [37]. The technique has been frequently used in several previous studies [38–41]. Although our tissue staining results were further supported by the upregulation of ERManI in hepatoma cell lines, it would be ideal to determine the level of upregulation in HCC tissues using a more quantitative method such as quantitative western blotting since immunohistochemistry is semi-quantitative, and can be affected by the antibody reactivity upon retrieved antigens.

The function of ERManI in regulating transformation phenotypes is not limited to PLC/PRF5 cells, because siRNA-mediated knockdown of ERManI also slows the proliferation of HepG2 cells (Figure S2). In addition, ERManI seems to function in a stoichiometric manner since further elevation of ERManI in both PLC/PRF5 and HepG2 cells increases cell proliferation (Figure S3). Irrespective of these observations, we currently do not know the exact mechanism by which ERManI regulates proliferation and migration of hepatoma cells. Although it is predicated to function on all N-glycosylated proteins, knockdown of ERManI does not change the global protein N-glycosylation pattern, both intracellularly and extracellularly. In addition, knockdown of ERManI does not affect endogenous A1AT, whose altered expression and modification have been implicated in HCC. Based on these observations, we believe that the role of ERManI in regulating glycoprotein quality control may be limited to a subset of misfolded proteins such as mutant A1AT, and therefore does not affect the all protein substrates for the ER proteostasis network. Hence, it is unlikely that ERManI regulates cell proliferation/migration during tumorigenesis by affecting the global glycoprotein quality control system. We recently identified a physical interaction between ERManI and γ-COP, a subunit of the COPI coatomer responsible for Golgi-to-ER protein trafficking [31]. Interestingly, previous studies have shown that γ-COP binds to Cdc42 and is necessary for the transformation potency for the later molecule [51]. ERManI knockdown does not significantly affect the steady state level of γ-COP [31]. However, whether the interaction between γ-COP and Cdc42 is affected upon ERManI knockdown remains to be investigated.

In summary, the current study has revealed a novel post-transcription regulatory mechanism for human ERManI and provided the first lines of evidence that the molecule can exert a positive role in HCC development. These findings provide a novel link between the newly discovered Golgi protein quality control component and cancer. In addition, the new data support the possibility that, as a target for miR-125b, ERManI might be involved in the development of many other types of cancer.

## Materials and Methods

### cDNA constructs and siRNAs

Negative control miRNA, and miRNA mimics for miR-125a and miR-125b were purchased from Life Technologies (Grand Island, NY). ERManI cDNA was generated previously [23]. To generate luciferase reporter, the 3’UTR of ERManI was amplified from the ERManI cDNA using a forward primer 5’-aatctagaggtggatggctgctg-3’ and a reverse primer 5’- cctctagaagagcaaatcaacttttatctcc-3’, and cloned into pGL3-control vector (Promega, Madison, WI) using XbaI and SalI restriction sites. The above luciferase reporter was used as a template to generate the “seed” region mutation using the QuikChange site-directed mutagenesis kit (Stratagene, La Jolla, CA). The mutagenic primers are designed using the QuikChange primer design program from Stratagene, and the sequences are available upon request. ERManI siRNAs used in this study were purchased from Life Technologies and the sequences were described previously [23].

### Antibodies

Anti-ERManI monoclonal antibodies were generated previously [23]. Anti-actin antibodies were purchased from Sigma Aldrich (St. Louis, MO). Anti-PARP antibody and anti-eIF2α antibodies were purchased from Cell Signaling Technology (Danvers, MA). Anti-phospho-eIF2α antibodies were purchased from Invitrogen (Carlsbad, CA). Anti-XBP1 antibodies were purchased from Santa Cruz biotechnology (Santz Cruz, CA). Anti-BiP monoclonal antibody was purchased from BD Biosciences (San Jose, CA). Anti-human alpha1-antitrypsin antibodies were purchased from MP Biomedicals (Solon, OH). Anti-LC3 antibodies were purchased from Novus Biologicals (Littleton, CO). Anti-ATF6 antibodies were purchased from Imgenex (San Diego CA).

### Cell lines and tissues

PLC/PRF5 cells [23,52] were cultured in MEM (VWR) supplemented with 10% FBS and 1% ampicilin/streptomycin. HeLa cells were cultured in Dulbecco’s Modified Eagle’s Medium (DMEM) (VWR, Radnor, PA) supplemented with 10% fetal bovine serum (FBS) (Gemini Bio-Products, West Sacramento, CA) and 1% ampicillin/streptomycin (Invitrogen). MCF7 cells were cultured in RPMI1640 (Mediatech) supplemented with 10% fetal bovine serum and 1% ampicillin/streptomycin. Normal human liver tissues were obtained from Liver Tissue Cell Distribution System (LTCDS, University of Minnesota and University of Pittsburgh, #N01-DK-7-0004C/HHSN267200700004C).

### Transient transfection and western blotting

PLC/PRF5 cells were plated into 60mm dishes and allowed to reach 60% confluence by the time of transfection. Each miRNA was transfected at indicated amount into the cells using Difectin 3 (Panora Biotech, Sugar Land, TX). For transfection of luciferase reporters, HeLa cells grown in 24-well dishes were transfected with luciferase constructs, renilla plasmid, and/or miRNAs using lipofactamine2000 (Invitrogen). For western blotting, cells were lysed in lysis buffer containing 50-mM TrisHCl, 150-mM NaCl, 0.5% NP-40, 1-mM Na3VO4, 10-mM NaF, 2-mM PMSF, and equivalent amount of cell extracts were separated on SDS-PAGE, transferred onto nitrocellulose membrane, and followed by western blotting using appropriate antibodies.

### Luciferase assay

Transfected HeLa cells in each well of the 24-well dishes were lysed with 100µl of lysis buffer. 20µl of the cell extract was used for measuring luciferase and renilla activity on GloMax® 20/20 Luminometer (Promega) using the Dual-luciferase reporter assay system (Promega).

### Quantitative reverse transcription-PCR (RT-PCR) of miRNA

Total RNA was isolated from normal liver tissues and cell lines using mirVana^TM^ miRNA isolation kit (Applied biosystems, Foster City, CA) following the manufacturer’s instruction. 6ng of the total RNA was used for reverse-transcription using the Taqman microRNA reverse transcription kit (Applied biosystems) and RT primers provided by the TaqMan MicroRNA assays specific for miR-125a-5p and miR-125b (Applied biosystems). The resulted cDNA was then diluted with 1:15 ratio and the diluted cDNA was used for real-time PCR following the using the primers provided by the TaqMan MicroRNA assays specific for miR-125a-5p and miR-125b (Applied biosystems).

### Histological studies

Liver disease spectrum tissue microarray array (LV8011) was purchased from Biomax, Inc (Rockville, MD). The slide was stained with anti-ERManI monoclonal antibody 1D6 [23] in the pathology core facility in Baylor College of Medicine (Houston, TX). Each sample was given a proportion score (PS) and an intensity score (IS) by an experienced pathologist in the core. The PS and IS were used for statistical analysis using Mann-Whitney rank sum test. The images of the stained samples were acquired by Olympus BX1 system (Carl Zeiss, Thornwood, NY).

### Metabolic radiolabeling and immunoprecipitations

The procedure for [^35^S]-Met labeling, chase, as well as immunoprecipitations was described previously [23]. To isolate all glycoproteins, the cell extracts and mediums collected at each time point were incubated with 50µl of glycoprotein enrichment resin (Clontech, Mountain View, CA). After stringent wash, the bound proteins were eluted with SDS sample buffer and the proteins were resolved on SDS-PAGE followed by autoradiography.

### Measuring Cell growth

Transfected PLC/PRF5 cells were evenly seeded into 9 wells of 24-well dishes at 1x10^4^/well. After cultured for 24, 48, or 72-hrs, cells from three wells were detached by trypsinization and re-suspended in 1ml of culture medium. And the total number of cells in each well was determined using coulter counter (Beckman Coulter, Inc., Brea CA). The average number of cells in each well was used for calculating the cell number increase at each time points.

### Measuring cell migration and invasion

To measure cell migration, transfected PLC/PRF5 cells were evenly seeded into 9 wells of 24-well dishes at 5x10^4^/well and allowed to attach by culturing for 24hr. Two wounds were then made in both vertical and horizontal directions cross each well using a 10µl pipette tip, and the cells were continue cultured in fresh medium for additional 24hr or 48hr. At each time point, images along the longitude of the wounds were taking under phase contrast microscope and the areas of the wounds were measured using NIH imageJ software. To measure cell invasion, the upper well of the 24-well transwell (BD Biosciences, Bedford, MA) was pre-coated with 0.05mg/ml purified bovine collagen (Advanced Biomatrix, San Diego, CA) before seeded with 2.5x10^4^ of cells in serum free-medium. The upper wells were placed into the lower wells supplied with regular culture medium and cultured for 24hr. Subsequently, cells in the upper wells were removed using a cotton swab, and the wells were washed three times with PBS before being stained with 4% paraformaldehyde/0.1% Crystal violet (Sigma Aldrich, St. Louis, MO) for 10min and air dried. The filters were removed from the well and mounted on slides. The cells on three repeated filters were counted and the average numbers of the cells were used for analysis.

## Supporting Information

Table S1 **Pathological evaluation results of the liver tissue spectrum microarray.**
The tissue microarray slide containing 80 samples each with specific characterizations (position, sex, age, organ, pathology, disease grade, disease state, TNM score for classification of malignant tumors, and type of disease) was stained with the anti-ERManI antibody 1D6. A score for the percentage of stained cells (PS) and a score for the signal intensity (IS) were applied to each sample.
(XLSX)

Figure S1 **miR-125a suppresses ERManI expression at a high dose.**
The percentage of ERManI expressed in MCF7 cells transfected with indicated amount of miR-125a as compared to that transfected with equal amounts of control miRNA. The data were based on the densitometry measurement of the protein bands detected by western blotting.
(TIF)

Figure S2 **Downregulation of ERManI inhibits proliferation of HepG2 cells.**
Growth curve of HepG2 cells 72hr after transfection with control siRNA or ERManI siRNA #1. Error bars represent standard deviations from three replicates.
(TIF)

Figure S3 **Upregulation of ERManI promotes proliferation of hepatoma cells.**
**A**. Growth curve of PLC/PRF5 cells 72hr after transfection with empty vector or ERManI cDNA. Error bars represent the standard error of mean. B. Growth curve of HepG2 cells 72hr after transfection with empty vector or ERManI cDNA. Error bars represent standard error of the mean.
(TIF)
