# Correction: ERManI Is a Target of miR-125b and Promotes Transformation Phenotypes in Hepatocellular Carcinoma (HCC)

**DOI:** 10.1371/annotation/a8938c97-3002-45c3-bde5-91952a5fa4eb

**Published:** 2014-01-02

**Authors:** Shujuan Pan, Xiaoyun Cheng, Hongan Chen, Patricia D. Castro, Michael M. Ittmann, Anne W. Hutson, Susan K. Zapata, Richard N. Sifers

Reference 31 is incomplete. The corrected reference is:

Pan, S., Cheung, X. and Sifers, R.N. (2013) Golgi-situated ERManI contributes to the retrieval of ERAD substrates through a direct interaction with γ-COP. Molecular Biology of the Cell 24, 1111-1121. 

