# Supplementary figures and images for: ERManI Is a Target of miR-125b and Promotes Transformation Phenotypes in Hepatocellular Carcinoma (HCC)
Source: PLoS One. 2013 Aug 5;8(8):e72829. doi: 10.1371/journal.pone.0072829 (PMC3733964; doi:10.1371/journal.pone.0072829)

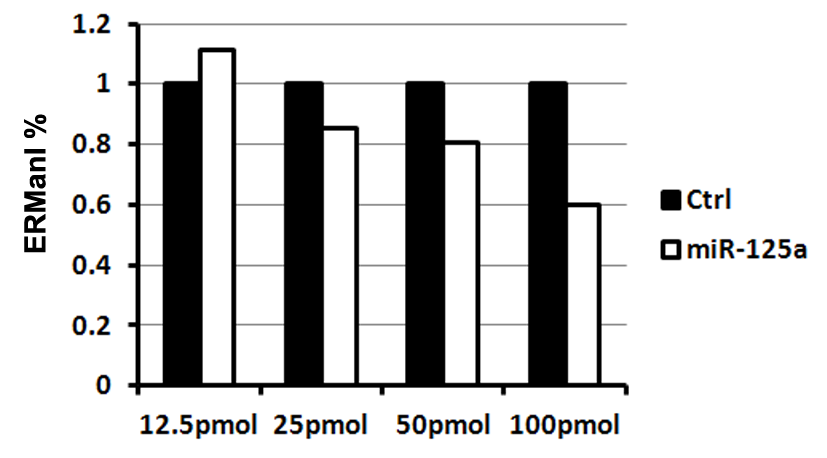

Supplement: Figure S1 — miR-125a suppresses ERManI expression at a high dose. The percentage of ERManI expressed in MCF7 cells transfected with indicated amount of miR-125a as compared to that transfected with equal amounts of control miRNA. The data were based on the densitometry measurement of the protein bands detected by western blotting. (TIF) [file pone.0072829.s002.tif]

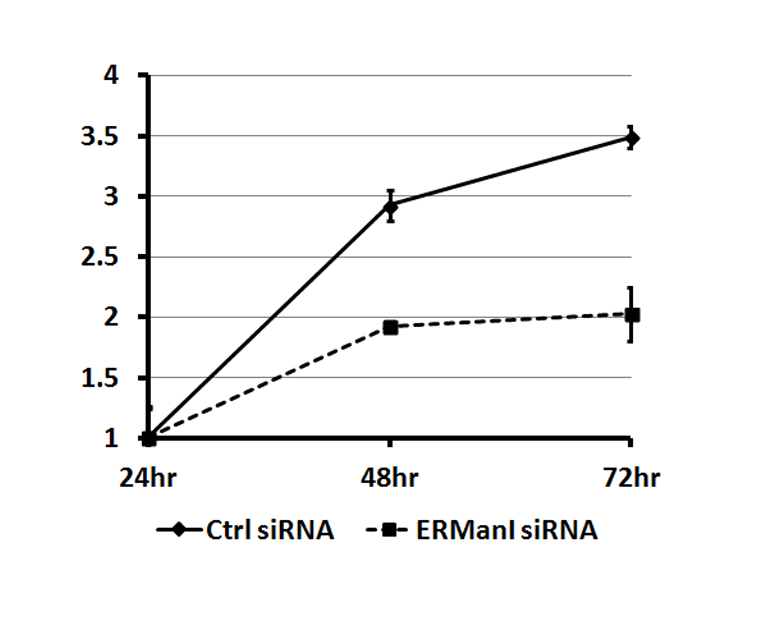

Supplement: Figure S2 — Downregulation of ERManI inhibits proliferation of HepG2 cells. Growth curve of HepG2 cells 72hr after transfection with control siRNA or ERManI siRNA #1. Error bars represent standard deviations from three replicates. (TIF) [file pone.0072829.s003.tif]

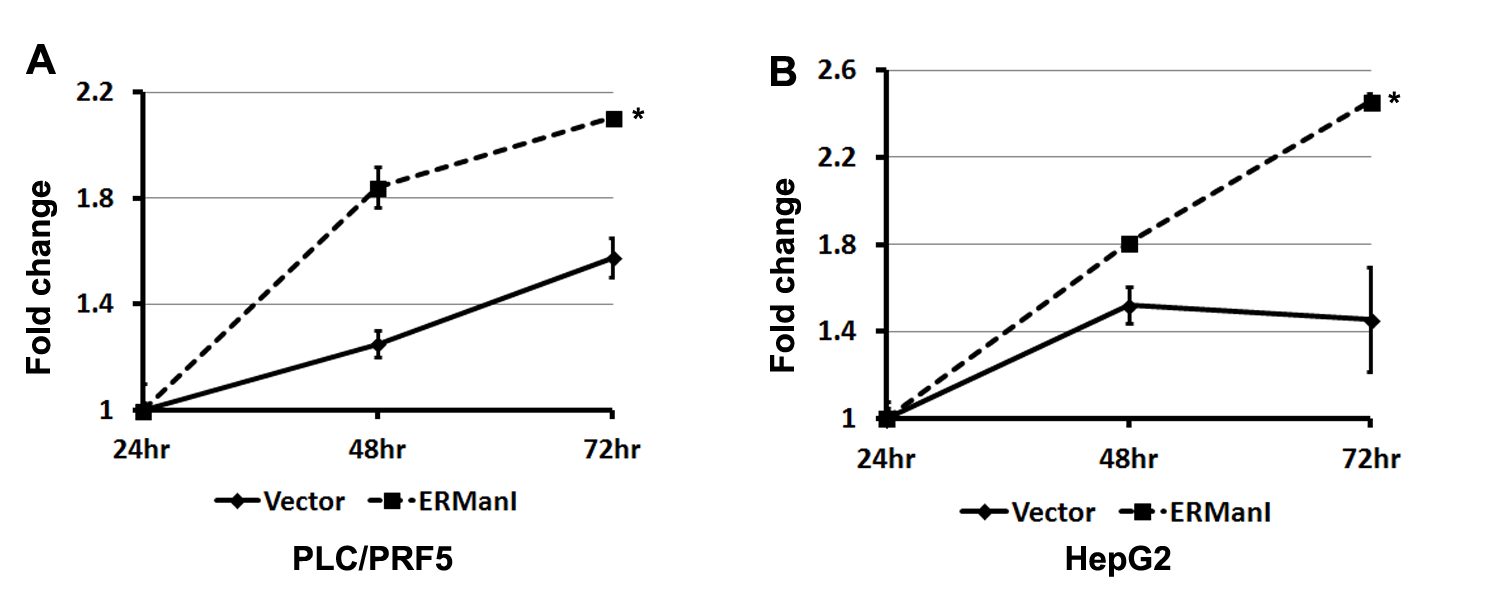

Supplement: Figure S3 — Upregulation of ERManI promotes proliferation of hepatoma cells. A. Growth curve of PLC/PRF5 cells 72hr after transfection with empty vector or ERManI cDNA. Error bars represent the standard error of mean. B. Growth curve of HepG2 cells 72hr after transfection with empty vector or ERManI cDNA. Error bars represent standard error of the mean. (TIF) [file pone.0072829.s004.tif]
